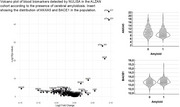# Study of blood biomarkers detected in NULISA to stratify the prospective multicenter ALZAN cohort of memory clinic patients according to ATN status, presence of synucleinopathy and diagnosis

**DOI:** 10.1002/alz70856_106853

**Published:** 2026-01-10

**Authors:** Sylvain Lehmann, Constance Delaby, Nicolas Pradeilles, Said Assou, Christophe Hirtz, Germain Busto, Brice Dupuy, Marie Duchiron, Mehdi Morchikh, Geneviève Barnier‐Figue, Florence Perrein, Audrey Gabelle, Cédric Turpinat, Snejana Jurici, Karim Bennys

**Affiliations:** ^1^ LBPC‐PPC, Université de Montpellier, INM INSERM, IRMB CHU de Montpellier, Montpellier, France, Montpellier, France; ^2^ MGX, Université de Montpellier, IRMB INSERM CHU de Montpellier, Montpellier, France, Montpellier, France; ^3^ CHU Montpellier CMRR, Montpellier, France; ^4^ CH Perpignan, Gériatrie, Perpignan, France; ^5^ CHU Nimes, Nimes, France; ^6^ Université de Montpellier, Memory Research and Resources center, department of Neurology, Inserm INM, F‐34000 Montpellier, France., Montpellier, France

## Abstract

**Background:**

The objective of this study in the multicenter prospective ALZAN cohort is to assess the performance of blood biomarkers for detecting biological processes based on the ATN classification and synuclein seed amplification assay (SAA), as well as for diagnosing Alzheimer's disease (AD) and related dementias, including frontotemporal dementia and Lewy body dementia.

**Method:**

The ALZAN cohort (NCT05427448) consists of over 400 patients seen in memory clinics at the Montpellier and Nîmes university hospitals and Perpignan hospital. As part of standard care, cerebrospinal fluid (CSF) biomarkers (Aβ40/42, Tau, pTau181) are measured. Clinical and biological data, including ApoE4 status and Mini‐Mental State Examination (MMSE) scores, are collected. Blood biomarkers are measured in plasma using the ultrasensitive multiplex NULISA approach, allowing relative quantification of 120 biomarkers of interest (https://alamarbio.com/). Biomarker performance is assessed by comparing patient groups stratified by core CSF AD biomarkers, SAA, and clinical diagnosis.

**Result:**

Among the 120 biomarkers measured using NULISA, those that remained statistically significant after adjustment for age, sex, body mass index (BMI), and ApoE4 status, considering multiple comparisons, were ranked in order of performance as follows:

**For brain amyloidopathy (57% of the population, based on CSF biomarkers)**: pTau_217>pTau_231>pTau_181>GFAP>MAPT>BACE1>SQSTM1>ANXA5>RUVBL2>TARDBP>NRGN.

**For neurodegeneration (49% of the population, based on total Tau in CSF)**: pTau_231>pTau_217>pTau_181>GFAP>MAPT>RUVBL2>TARDBP>IL18>ANXA5>NRGN>pTDP43_409>HTT>pSNCA_129>IL1B>PGK1>CD40LG>SOD1>FGF2>IL7>Oligo_SNCA>ARSA>PRDX6>PSEN1>SNCA>SQSTM1>UCHL1.

**For Alzheimer's disease (51% of the population, defined by clinico‐biological criteria)**: pTau_231>pTau_217>pTau_181>GFAP>MAPT>BACE1>TARDBP>ANXA5>NRGN>RUVBL2>pTDP43_409>pSNCA_129>IL18>SOD1>SNCA>NCA>SQSTM1>CRP>MDH1.

**For synucleinopathy (14% of the population, based on CSF SAA)**: No blood biomarkers showed significant differences.

**Conclusion:**

This study identifies among the 120 biomarkers many already known to be pathological in AD. However, it also highlights new potential targets, emphasizing the value of this multiplex approach. Further analysis combining multiple biomarkers (ratios) will be useful, along with investigations into the impact of confounding factors such as renal function and pre‐analytical treatment. Notably, none of these blood biomarkers were able to differentiate between SSA‐positive and negative cases.